# Exergaming as a Viable Therapeutic Tool to Improve Static and Dynamic Balance among Older Adults and People with Idiopathic Parkinson’s Disease: A Systematic Review and Meta-Analysis

**DOI:** 10.3389/fnagi.2015.00167

**Published:** 2015-09-07

**Authors:** Dale M. Harris, Timo Rantalainen, Makii Muthalib, Liam Johnson, Wei-Peng Teo

**Affiliations:** ^1^Centre for Physical Activity and Nutrition Research, School of Exercise and Nutrition Sciences, Deakin University, Burwood, VIC, Australia; ^2^Movement to Health (M2H) Laboratory, Euromov, University of Montpellier, Montpellier, France; ^3^Clinical Exercise Science Research Program, Institute of Sport Exercise and Active Living (ISEAL), Victoria University, Melbourne, VIC, Australia; ^4^The Florey Institute of Neuroscience and Mental Health, University of Melbourne, Melbourne, VIC, Australia

**Keywords:** Parkinson’s, balance, exergaming, older, adults

## Abstract

The use of virtual reality games (known as “exergaming”) as a neurorehabilitation tool is gaining interest. Therefore, we aim to collate evidence for the effects of exergaming on the balance and postural control of older adults and people with idiopathic Parkinson’s disease (IPD). Six electronic databases were searched, from inception to April 2015, to identify relevant studies. Standardized mean differences (SMDs) and 95% confidence intervals (CI) were used to calculate effect sizes between experimental and control groups. *I*^2^ statistics were used to determine levels of heterogeneity. 325 older adults and 56 people with IPD who were assessed across 11 ­studies. The results showed that exergaming improved static balance (SMD 1.069, 95% CI 0.563–1.576), postural control (SMD 0.826, 95% CI 0.481–1.170), and dynamic balance (SMD −0.808, 95% CI −1.192 to −0.424) in healthy older adults. Two IPD studies showed an improvement in static balance (SMD 0.124, 95% CI −0.581 to 0.828) and postural control (SMD 2.576, 95% CI 1.534–3.599). Our findings suggest that exergaming might be an appropriate therapeutic tool for improving balance and postural control in older adults, but more ­large-scale trials are needed to determine if the same is true for people with IPD.

## Introduction

Idiopathic Parkinson’s disease (IPD) is the second most common neurological disease worldwide, affecting approximately 1% of all older adults aged 65 years or older (Moore et al., 2005; Lees et al., 2009). People with IPD experience tremors, rigidity, slowness of movement, and gait and balance dysfunction (Dibble et al., 2004; Lees et al., 2009; Zettergren et al., 2011). The balance impairments, which progressively worsen over time, increase the risk of falls, fall-related injuries, and mortality (Bloem et al., 2001; Wood et al., 2002) found 68.3% of people with IPD fell at least once per year, while 50.5% were recurrent fallers (two or more falls per year), while Lindholm et al. (2015) found 31% people with IPD reported nearly falling in the previous 12 months. Medications, such as levodopa, are often the first line of treatment for IPD and have shown promise in improving motor function in the early stages of the disease (Singh et al., 2007; Lees et al., 2009). However, the long-term efficacy of these treatments are poor, and levodopa therapy does little to preserve balance during the more advanced stages of the disease when balance is typically most affected (Bloem et al., 1996; Hely et al., 2005).

Exergaming, in this instance, is a term used to describe computer games that require players to physically move in response to game demands and/or an on-screen avatar (usually constructed from a sensor that records the player’s physical characteristics). Examples of exergaming models include interactive 3-Dimensional gaming modules, such as the Nintendo Wii Fit and the X-Box Kinect. Exergaming has the potential to facilitate balance improvements, including reducing standing center of pressure (CoP) variability (Rendon et al., 2012; Toulotte et al., 2012; Bieryla and Dold, 2013; Wuest et al., 2014), in a home-based setting for older adults (Miller et al., 2014) and for people recovering from spinal cord injury, brain injury (Betker et al., 2007 or stroke (Hung et al., 2014) who have severe balance impairments.

The wide variety of commercially available exergames and the scope of exergaming intensity levels allow for interventions to be tailored to target-specific aspects of balance (Kahlbaugh et al., 2011; Chao et al., 2014). Additionally, the range of exergames available to the consumer can stimulate diversity within training programs, which can aid in creating a fun and engaging “virtual” atmosphere (Barry et al., 2014; Ravenek et al., 2015). Such an environment is conducive to high levels of exercise adherence and participant motivation (Ravenek et al., 2015), which is likely to enhance the efficacy of the training. Duque et al. (2013) and Wuest et al. (2014) presented high interventional compliance rates (97 and 100%, respectively) in their exergaming interventions, though like many of the current exergaming studies, long-term exercise adherence was not reported. Moreover, some games include demanding cognitive and/or motor tasks that may be beyond the capability of people with IPD (Dos Santos Mendes et al., 2012). This may cause frustration and in turn cause people with IPD to avoid the games altogether (Dos Santos Mendes et al., 2012). This highlights the importance of individualized rehabilitation programing to effectively improve balance (Dos Santos Mendes et al., 2012).

Previous exergaming studies have attempted to quantify static and dynamic balance using the Berg balance scale (BBS) (Berg, 1989) and the Timed Up-and-Go test (TUG), for older adults (Agmon et al., 2011; Bateni, 2012; Franco et al., 2012), and for people with IPD (Zettergren et al., 2011; Mhatre et al., 2013). These tests are low-cost, simplistic assessments that are relatively quick to administer and convenient in the clinical setting. However, the subjective nature of these tests and their questionable sensitivity to detect slight changes in CoP deviation compared to posturographic technologies (Black, 2001) highlight the problematic use of these tests. Furthermore, of the current available exergaming randomized control trials (RCTs), there appears to be limited posturographic reporting of static or dynamic balance for older adults, and we know of only one RCT that has used posturography to report balance changes following exergaming for people with IPD (Yen et al., 2011).

Exergaming has the potential to increase exercise adherence, balance confidence, and exercise enjoyment (Barry et al., 2014; Miller et al., 2014; Ravenek et al., 2015), but it is unclear if it can improve the balance of people with IPD (Agmon et al., 2011; Meldrum et al., 2012; Holmes et al., 2013; Barry et al., 2014). There is limited evidence to suggest exergaming is an efficacious rehabilitation method for balance and postural issues experienced by older people and people with IPD. Therefore, we intend to systematically review exergaming RCTs and use a meta-analytical approach to compare the effects of exergaming on the balance of older adults and people with IPD.

## Methods

### Search strategy

**Figure 1:**
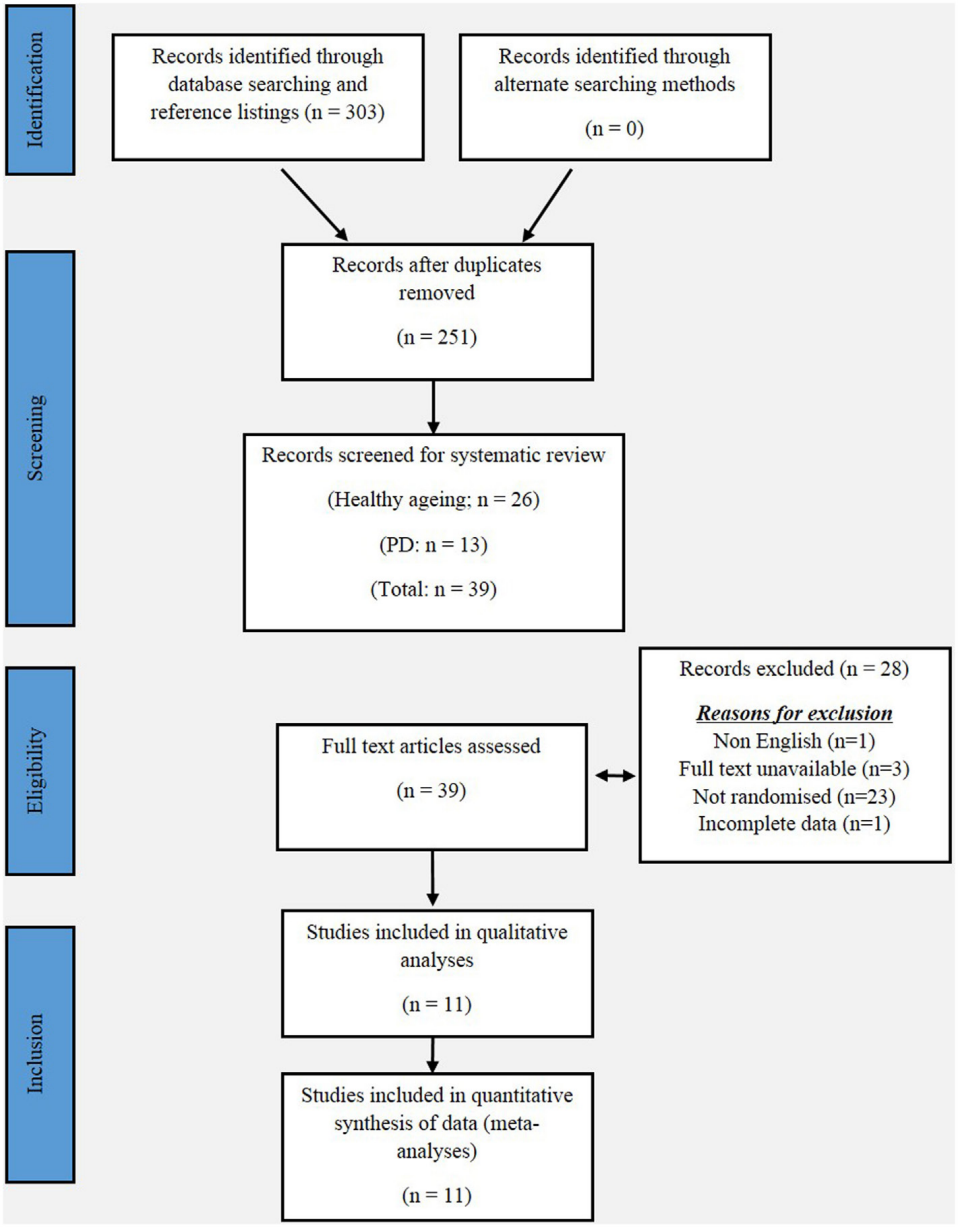
**PRISMA flow chart for the selection of studies included in this meta-analyses**.

This review has been informed by the PRISMA statement. The following electronic databases were searched from their inception to April 2015: PubMed, MEDLINE, PsycINFO, Embase, Google Scholar, and Scopus. The following keywords were used in combinations: Parkinson, Parkinson’s disease, Parkinsonism, exergaming, gaming, virtual reality gaming, series gaming, gait, balance, aged, and elderly. Additionally, the reference lists of the included studies were also searched. Figure 1 shows a flow diagram of the processing of search results from initial searches to the final included studies.

### Inclusion criteria

To determine the eligibility of each study, the title and abstract were screened independently by a single reviewer (Dale M. Harris). Studies were included if (1) it was printed in full English text, (2) the aim of the study was to examine the effects of exergaming on the static and dynamic balance of older adults and/or people with IPD, (3) the target population was aged 65 years or older, (4) the main intervention was exergaming, serious gaming, or virtual reality gaming, and (5) the effects of the gaming intervention were compared to either a control group, or an alternative training/rehabilitation intervention, including other forms of exercise, balance training, or scheduled general physical activity. All studies included were randomized, and only published articles (including articles in press) were included. All duplicate articles were removed.

### Quality assessment of studies

Data were extracted by an independent reviewer (Dale M. Harris), and revised by a second reviewer (Wei-Peng Teo). The methodological quality of each study was independently assessed by two reviewers (Liam Johnson and Timo Rantalainen) using the physiotherapy evidence database (PEDro) scale (ranging from 0 to 10 points). The PEDro scale is an assessment tool for evaluating methodological quality of RCTs conducted in the field of physiotherapy with fair-to-good reliability (Maher et al., 2003). Any disagreements in scores were resolved by discussion between reviewers, with the judgment of the primary author (Dale M. Harris) being sought if consensus could not be reached. All scores assigned to each study were agreed upon by unanimity and are presented in Table 4.

### Selection of outcome measures

For the purpose of our meta-analysis, we categorized the balance outcome measures into static and dynamic assessments. We chose the BBS (Berg et al., 1991) as our measure of static balance. The BBS (Berg et al., 1991) consists of 14 balance-challenging tasks, whereby the participants base of support remained fixed, or moved marginally, and each task is scored on a 0–4 scale (maximum score = 56). A higher score is indicative of better balance (Thorbahn and Newton, 1996).

We have also used postural control (PC) measures as assessments of static balance because they required no (or limited) movement of the participants base of support. Studies employing motion capture technology that were capable of quantifying the amount of postural sway circumference or CoP deviation, including posturographic measures, force plates, digital sway meters, virtual avatars, or Wii balance-board technology, were included as measures of PC.

We identified the TUG test, which involves the purposeful movement of the participants base of support, as a measure of dynamic balance. The TUG measures the total time (seconds) that an individual takes to rise from a chair, walk at a fast pace for 3-m, turn around, walk back and sit on the same chair (Podsiadlo and Richardson, 1991).

### Data synthesis and analysis

A random effects meta-analysis was conducted with MedCalc Statistical Software v14.12.0 (MedCalc Software bvba, Ostend, Belgium; http://www.medcalc.org: 2015). Continuous measures extrapolation yielded standardized mean difference (SMD) results, which were used as a measure of effect size, along with 95% confidence intervals (CI). Heterogeneity across studies was tested based on *I*^2^ statistics, which indicates the percentage of variance that is attributable to study heterogeneity. Studies with *I*^2^ < 40% was considered to have low heterogeneity, *I*^2^ = 40–75% was considered to have moderate heterogeneity, and *I*^2^ > 75% was considered to have high heterogeneity. Fisher’s method of combining *p*-values was applied to test for overall effects for each outcome measure.

## Results

### Study selection

**Figure 2:**
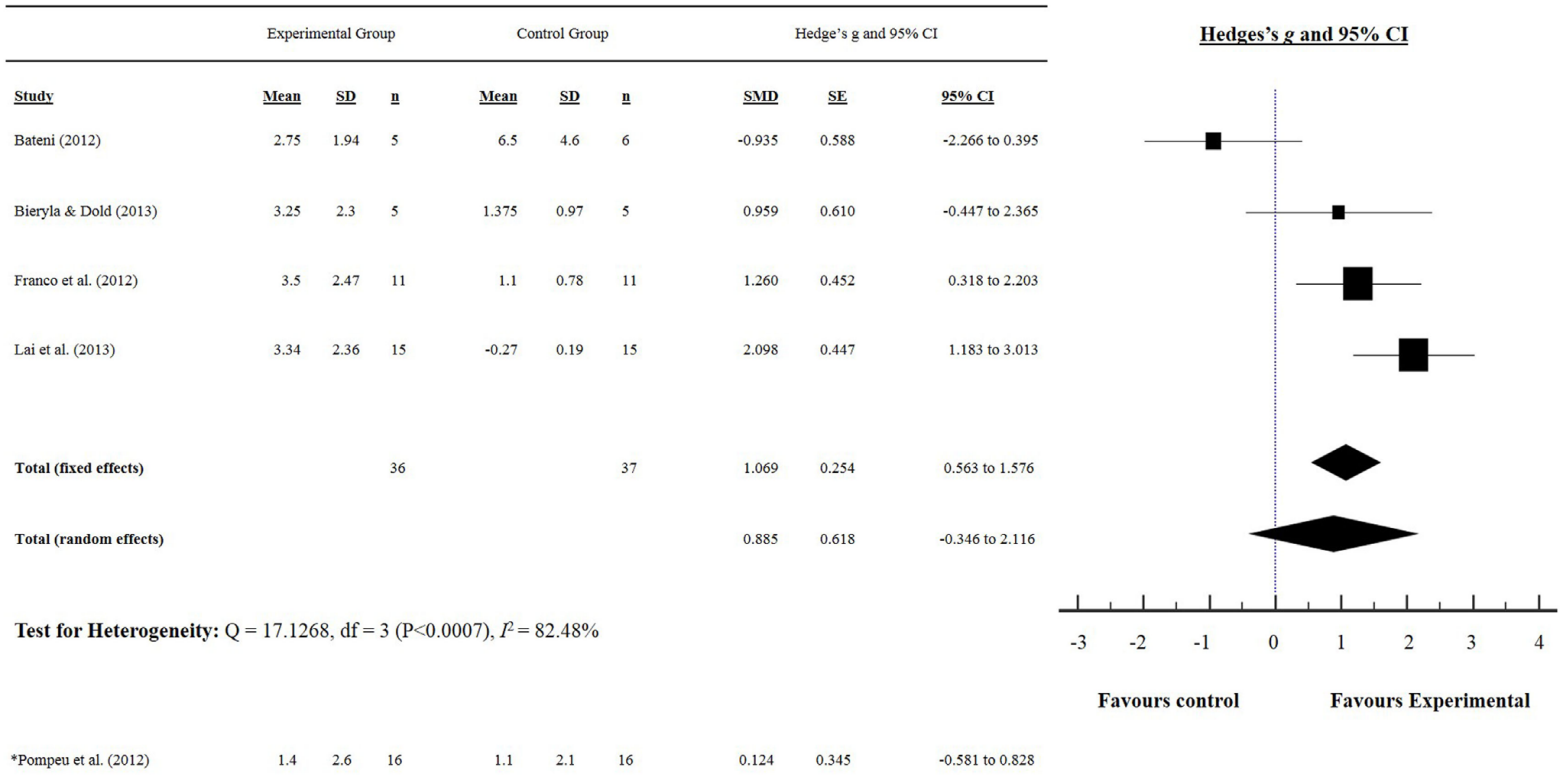
**Static balance: Berg balance scale score**. (*) indicates only IPD study that used the BBS as a measure of static balance.

**Figure 3:**
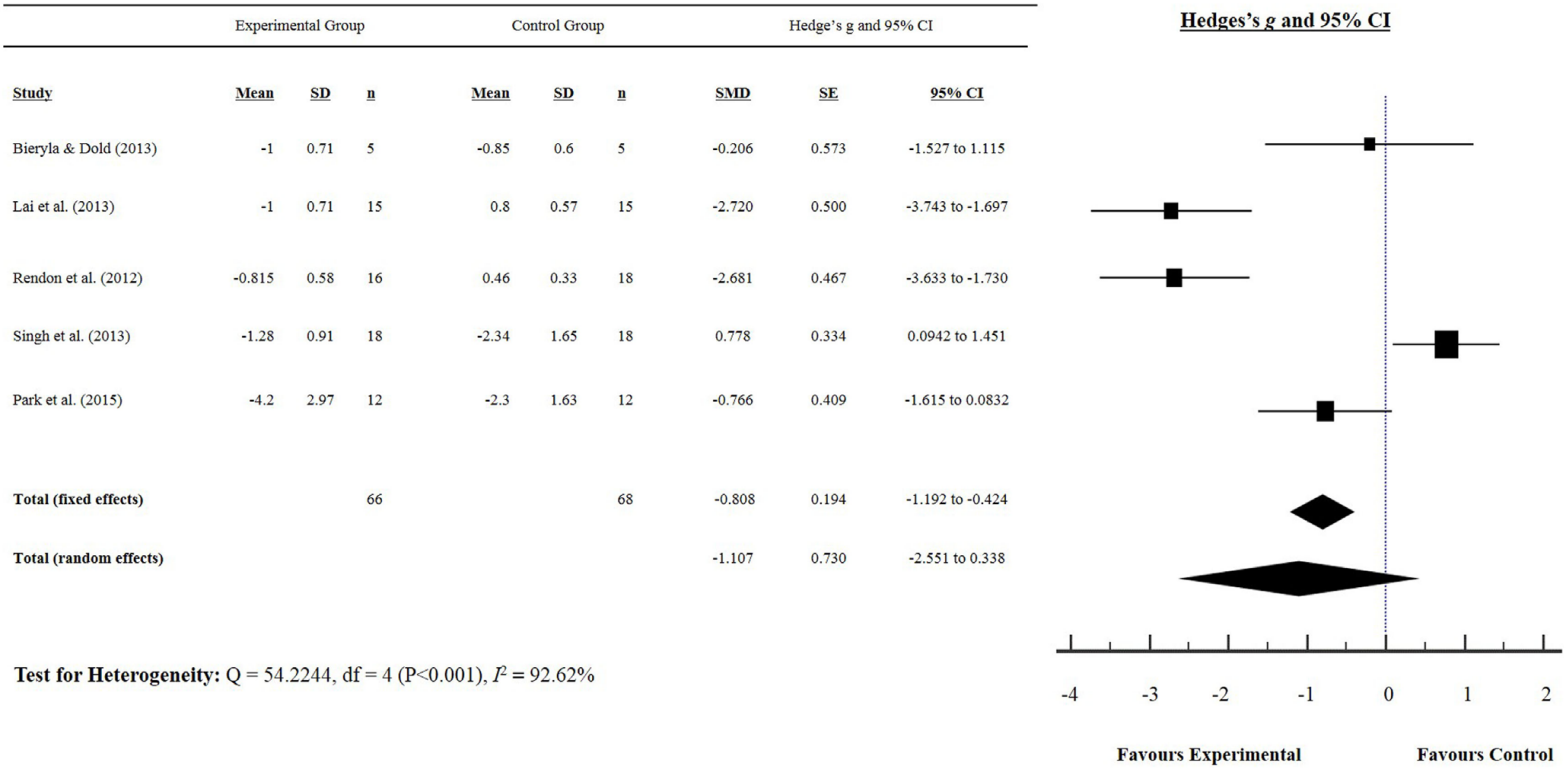
**Dynamic balance: timed up and go test score for older adults**.

Our initial search yielded 303 articles. Following screening of the title and abstract, and removal of duplicates, 11 studies were included in our meta-analysis, and are summarized in Tables 1 and 2. Five studies reported a measurement of static balance (BBS) (Bateni, 2012; Franco et al., 2012; Pompeu et al., 2012; Bieryla and Dold, 2013; Lai et al., 2013) (Figure 2), and five studies reported a measurement of dynamic balance (TUG) (Rendon et al., 2012; Bieryla and Dold, 2013; Lai et al., 2013; Singh et al., 2013; Park et al., 2015) (Figure 3). Seven studies reported a measurement of PC (Yen et al., 2011; Bateni, 2012; Toulotte et al., 2012; Kim et al., 2013; Lai et al., 2013; Singh et al., 2013; Park et al., 2015) (Table 3).

**Table 1:** **Characteristics of exergaming randomized controlled trials studies among people with IPD**.

Author	Sample size, mean age (years) **±** SD	Hoehn and Yahr stage	Medications	Duration (weeks)	Main outcome assessments	Intervention groups	Control group
Pompeu et al. (2012)	*n* = 32 VR and CON 67.4 ± 8.1	1 and 2	Levodopa therapy-outcome measures and training interventions were performed during “on” phase	7	UPDRS-II	VR and cognitive training + global exercises (1 h, 2 sessions/week)	TBT
					Static balance	• 10 min of warming, stretching and active exercises; 10 min of resistance exercises for limbs; and 10 min of exercises in diagonal patterns for trunk, neck and limbs	• 10 exercises (5 per session, 2 trials of each) that were equivalent to the motor demands of the Wii training group, but without the provision of external cues, feedback, and cognitive stimulation
					Dynamic balance	• 10 min dynamic balance: Table Tilt, Tilt City, Soccer Heading and Penguin Slide	
						• 10 min static balance: rhythm parade, obstacle course, basic step, and basic run	
Yen et al. (2011)	*n* = 42 VR 70.4 ± 6.5; TBT 70.1 ± 6.9; CON 71.6 ± 5.8	2 and 3	Levodopa therapy-outcome measures and interventions were performed during “on” phase	6	Postural control	VR balance training (30 min, 2 sessions/week)	NI
					Verbal reaction time	• 10 min warm up	
						• VR training: 10 min of a 3D ball-rolling game and 10 min of indoor-outdoor virtual activities	
						TBT	
						• 10 min warm up, 20 min of TBT	
						• Static stance: participants stood on pieces of foam with eyes open or closed for approximately 60 s difficulty was increased by adding more foam pieces and reducing the base of support	
						• Dynamic weight shifting: a ball was thrown at patients from multiple directions, ball was caught after stepping forward and squatting	
						• Addition of a tilt board was used as external perturbation to facilitate postural reflexes under both static and dynamic conditions	

**Table 2:** **Characteristics of exergaming randomized controlled trials studies among older adults**.

Author	Participant number mean age (years) **±** SD	Duration (weeks)	Main outcome assessed	Intervention groups	Control group
Bateni (2012)	*n* = 8 VR 79 ± 13; PA 72 ± 12; COMBI 68 ± 14	4	Static balance	VR (3 sessions/wk)	NI
				• Wii Fit games: Ski Slalom, Ski Jump and Table Tilt	
				PA (3 sessions/wk)	
				• Standard strength, balance and postural exercises	
				COMB (3 sessions/wk)	
				• Combination of both physical therapy and Wii Fit games	
Bieryla and Dold (2013)	*n* = 12 VR 82.5 ± 1.6; CON 80.5 ± 7.8	3	Static balance	VR (30 min, 3 sessions/wk)	NI
				• Training consisted of: half-moon, chair, warrior, torso twists, soccer heading and ski jump	
				• All maneuvers were chosen from the yoga, aerobic and balance game modes	
Franco et al. (2012)	*n* = 32 VR 79.8 ± 4.7; CON 76.9 ± 6.3	3	Static balance Dynamic balance Health and wellbeing	VR (10–15 min, 5 separate sessions) • Wii Fit group: received Wii Fit balance training and completed supplemental home exercises	Completed exercises from the MOB Program, administered by the staff exercise physiologist
Kim et al. (2013)	*n* = 32 VR 68.28 ± 3.7; CON 65.83 ± 3.7	8	Hip muscle strength Ground reaction force	VR (3 sessions/wk) • “Your Shape Fitness Evolved” software: includes movements derived from exercise programs based on Tai Chi and yoga • Bilateral shoulder abduction, and single-leg abduction • Abduction of the right (or left) arm to shoulder level with elbow flexion at 90°, and abduction of the ipsilateral leg with knee flexion at 90° • Abduction of the arms to shoulder level and flexion of both knees at 90° • Crossed arms in front of chest in a standing position with the feet splayed outward and the knees flexed at 90°	NI
Lai et al. (2013)	*n* = 30 VR 70.6 ± 3.5; CON 74.5 ± 4.7	6	Functional performance Static balance Stability confidence Postural control XMSS stepping test	VR (3 sessions/wk) • Subjects performed following movements: quiet stance, sitting-to-standing, shifting weight and reaching, turning in place, standing on one leg, and maintaining a tandem stance • Virtual reality XMSS stepping test procedures	NI
Park et al. (2015)	*n* = 24 VR 66.55 ± 8.1; BE 65.2 ± 7.9	8	Static balance	VR (30 min, 3 sessions/wk)	BE (30 min, 3 sessions/wk)
			Dynamic balance	• Participants spent 10 min on each of the Wii fit balance games including soccer heading, snowboard slalom, and table tilt	20 min bouncing, pelvic tilting laterally, pelvic tilting anterior-posterior, and the pelvic circling while sitting on an exercise ball
					10 min tilting the body to the right and left sides while putting their feet on the ball in a supine position, bending the knees and performing a hamstring bridge
Rendon et al. (2012)	*n* = 40 VR 85.7 ± 4.3; CON 83.3 ± 6.2	6	Dynamic balance Static balance Balance confidence	VR (35–45 min, 3 sessions/wk) • Lunges • Single-leg extensions • Twists	NI
Singh et al. (2013)	*n* = 36 VR 61.12 ± 3.72; CB 64 ± 5.88	6	Agility	VR (40 min, 2 sessions/wk)	TBT prescribed by Seidler and Martin (1997)
			Dynamic balance	• Ski slalom	
			Functional mobility	• Table tilt	
			Postural control	• Penguin slide	
				• Soccer heading	
				• Tight rope walk	
				• Perfect 10	
				• Tilt city	
Toulotte et al. (2012)	*n* = 36 VR 72.2 ± 8.6; CON 71.8 ± 8.0	20	Static balance Dynamic balance “Wii Fitness”	PA (1 h, 1 sessions/wk) • Exercises to increase step length, step height, the mobility of the cervical rachis and ocular mobility in order to develop muscular strength, proprioception, flexibility, static balance, and dynamic balance with eyes open and eyes closed VR (1 h, 1 session/wk) • Heading soccer, ski jumping, yoga, downhill skiing, game balls, and tight rope walker COMBI (30 min PA, 30 min VR, 1 session/wk) • Same PA and Wii Fit exercises as above, with half the repetitions of both groups	NI

*PA, physical activity; NR, not reported; NI, no intervention; TBT traditional balance training; VR, virtual reality; COMB, combination of both; UPDRS, unified Parkinson’s disease rating scale; MOB, matter of balance; BE, ball exercise; wk, week*.

**Table 3:** **Description of studies assessing postural control in older adults**.

Study	Assessment of postural control	VR intervention (*n*)	Control (*n*)	Total (*n*)	SMD	SE	95% CI	*t*	*p*
Bateni (2012)	Wii Fit game (Bubble Test) pre-post score: requires participants to maneuver their center of body mass in a controlled manner within a VR environment	5	6	11	−0.960	0.590	−2.294 to 0.375		
Kim et al. (2013)	Ground reaction force plate: backward step test with eyes open	18	14	32	1.412	0.390	0.617–2.208		
Lai et al. (2013)	Catsys 2000 system: a platform with 3 orthogonal strain gage devices	15	15	30	1.487	0.404	0.660–2.315		
Park et al. (2015)	Static postural sway length (mm) measurement system: “Biorescue”	12	12	24	0.636	0.405	−0.203 to 1.475		
Singh et al. (2013)	Intelligent balance board: combined scores to obtain an overall performance rating score	18	18	36	1.328	0.362	0.593–2.063		
Toulotte et al. (2012)	Estimated a percentage of center of gravity score by using “Wii Fit” balance assessment VR tool	9	9	18	1.578	0.520	0.475–2.681		
Total (fixed effects)		77	74	151	0.826	0.174	0.481–1.170	4.738	<0.001
Total (random effects)		77	74	151	0.714	0.454	−0.184 to 1.611	1.571	0.118

### Participant characteristics

In total, 225 older adults (experimental = 115, control = 110) and 56 people with IPD (experimental = 28, control = 28) were assessed across 11 studies. The mean age ±SD for the older adults was 73.32 ± 7.35 (experimental 73.97 ± 8.19 years, control 72.67 ± 6.84 years), while the mean age for people with IPD was 69.8 ± 2.16 (experimental 68.9 ± 2.12, control 71.6 ± 5.8). The mean duration of IPD was 6.9 ± 1.27 years, and disease severity, as assessed by the Hoehn and Yahr scale (Hoehn and Yahr, 1998), ranged from 1 to 3 (mild to moderate disease severity).

### Methodological quality

The scores for each criterion using the PEDro scale are presented in Table 5. The mean score for all 11 trials was 5.3 ± 1.5. Across the 11 studies, neither the participants nor the therapists administering the program were blinded to the intervention. Only one study concealed the allocation of all participants, used blinded assessors, and employed intention-to-treat analysis (Yen et al., 2011).

### Static balance: Berg balance scale

The effects of exergaming on static balance were examined by pooling post-intervention data from the five studies that reported BBS results from older adults and people with IPD (experimental *n* = 52, control *n* = 53) (Figure 2). Overall, the pooled results showed a significant improvement in static balance for older adults, as evidenced by increased BBS scores post-intervention (SMD 1.069, 95% CI 0.563–1.576). Pompeu et al. (2012) was the only study to examine the effect of exergaming on the balance of people with IPD and showed a favorable effect (i.e., an improvement in BBS score) (SMD 0.124, 95% CI −0.581 to 0.828). Only Bateni (2012) showed a non-favorable effect (i.e., a reduction in BBS score) for the exergaming intervention group (SMD −0.935, 95% CI −2.266 to 0.395) of older adults.

### Dynamic balance: Timed up and go test

The effects of exergaming on dynamic balance were examined by pooling post-intervention data from the five studies that reported TUG test results of older adults and people with IPD (experimental *n* = 66, control *n* = 68) (Rendon et al., 2012; Bieryla and Dold, 2013; Lai et al., 2013; Singh et al., 2013; Park et al., 2015) (Figure 3). Our findings showed a significant improvement in TUG (SMD −0.808, 95% CI −1.192 to −0.424), as indicated by a negative score (i.e., reduction in TUG time). Singh et al. (2013) was the only study to show a non-favorable effect following a 6-week exergaming intervention for older adults (SMD 0.778, 95% CI 0.0898–1.466).

### Postural control

Tables 3 and 4 summarize each study and their effect sizes regarding exergaming and PC in older adults (Table 3) and people with IPD (Table 4). Collectively, our results suggest that exergaming improves PC in older adults (SMD 0.826, 95% CI 0.481–1.170). Only Bateni (2012) reported a non-favorable effect following a 4-week exergaming and physical therapy training intervention for older adults (SMD −0.960, 95% CI −2.294 to 0.375). Yen et al. (2011) was the only study to investigate exergaming and PC of people with IPD, and found a significant improvement in PC for people with IPD (SMD 2.576, 95% CI 1.534–3.599).

**Table 4:** **Description of the study assessing postural control in people with IPD**.

Study	Assessment of postural control	VR intervention (*n*)	Control (*n*)	Total (*n*)	SMD	SE	95% CI
Yen et al. (2011)	Mean equilibrium scores from six sensory organizational tests	14	14	28	2.567	0.502	1.534–3.599

**Table 5:** **PEDro scale of quality for eligible randomized controlled trials**.

Study	Random allocation	Concealed allocation	Similar at baseline	Subjects blinded	Therapists blinded	Assessors blinded	**<**15% dropout	Intention-to-treat analysis	Between group comparisons	Point measures and variability data	Total
Bateni (2012)	1	1	1	0	0	0	1	0	0	1	5
Bieryla and Dold (2013)	1	0	1	0	0	0	0	0	0	1	3
Franco et al. (2012)	1	1	1	0	0	0	1	0	1	1	6
Kim et al. (2013)	1	0	1	0	0	0	1	0	1	1	5
Lai et al. (2013)	1	0	0	0	0	1	1	0	0	1	4
Park et al. (2015)	1	0	1	0	0	0	1	0	1	1	4
Pompeu et al. (2012)	1	1	1	0	0	1	1	0	1	1	7
Rendon et al. (2012)	1	0	0	0	0	1	1	1	1	1	6
Singh et al. (2013)	1	0	1	0	0	1	1	0	1	1	6
Toulotte et al. (2012)	1	1	0	0	0	0	1	0	0	1	4
Yen et al. (2011)	1	1	1	0	0	1	1	1	1	1	8

## Discussion

This study is the first to cohesively present the effects of exergaming on the balance of older adults and people with IPD. Our systematic review of the literature found limited robust evidence for the effects of exergaming on the balance of older adults and people with IPD. Of the RCTs included in our meta-analysis, the methodologies are not comprehensive enough to definitively elucidate if exergaming affects the balance of older adults or people with IPD. Our meta-analysis showed that no RCTs used the TUG as a measure of dynamic balance of people with IPD, and only two RCTs measured static balance using the BBS (Pompeu et al., 2012) or PC measures (Yen et al., 2011) for people with IPD. However, we found exergaming improves the static (BBS and PC measures) and dynamic (TUG) balance of older adults, despite the variability in PC measures used. Therefore, while our meta-analysis might suggest that exergaming can improve the balance of older adults, it remains largely unknown if the same is true for people with IPD due to a lack of sufficient evidence.

Only Bateni (2012) and Singh et al. (2013) found the static and dynamic balance of older adults was not improved by exergaming. An explanation for this could arise from their respective methodologies; the control groups for both studies included exercise interventions that specifically targeted balance (i.e., one-leg standing, free leg swinging, and moving objects), which may have contributed to the lack of effects found. These results might indicate that exergaming can improve balance as much as balance-specific training among older adults, which has previously been reported (Bateni, 2012; Franco et al., 2012; Toulotte et al., 2012; Singh et al., 2013).

It is beyond the scope of this study to determine the mechanisms responsible for the improvements in balance indicated by our meta-analysis. However, we can speculate that exergaming might provide a novel setting conducive to enhancing an individual’s ability to learn and perform new skills. For people with IPD who experience difficulties learning new skills (Scandalis et al., 2001; Dos Santos Mendes et al., 2012), the stimulus provided by exergaming might be strong enough to induce implicit learning (Mirelman et al., 2011). Additionally, immediate biofeedback of performance may also account for the augmented balance improvements of older adults (Bisson et al., 2007; Heiden and Lajoie, 2010; Franco et al., 2012; Cho et al., 2014; Wuest et al., 2014). Caudron et al. (2014) postulated that visual immersion provides a strong biofeedback cue for improving PC for people with IPD, although the effects of biofeedback on the balance of people with IPD is not clear. However, speculation of the opposite that certain exergames considered too challenging (i.e., exergames requiring dual tasking or decisive movements) might impair balance improvements for people with IPD has also been suggested (Dos Santos Mendes et al., 2012; Galna et al., 2014). Moreover, many interventions conducted in the clinical setting are unlikely to be replicated in the home setting, highlighting the importance of selecting appropriate exergames so that people with IPD can safely and effectively perform the tasks from home.

We have identified that static balance, indicative of PC changes, among older adults can be improved by exergaming, and Yen et al. (2011) demonstrated improvements in the PC of people with IPD. Interestingly, there were inconsistencies in effect sizes seen in both IPD studies compared with the pooled effects of older adults, with Yen et al. (2011) having a much larger effect size compared with Pompeu et al. (2012). To provide some explanations, Pompeu et al. (2012) contrasted an exergaming group against a traditional balance training group, while Yen et al. (2011) did not adopt an intervention for their control group providing a sound reason for the disparate results. Yen and colleagues used a combination of exergaming and traditional balance training in their intervention group. Furthermore, it is likely that the study by Yen and colleagues used participants that are between Hoehn–Yahr stages II and III, which may have allowed for the larger balance effects seen in Yen et al. (2011). Finally, IPD participants in both studies were using medication at the time of intervention, which can improve functional balance in the early stages of the disease (Nova et al., 2004), potentially reducing the effects of exergaming on balance in Pompeu et al. (2012).

The use of simplistic assessments to quantify static and dynamic balance presents as a limitation in the current exergaming literature, with posturography not commonly used. Posturography provides a more valid tool for effectively measuring static and dynamic balance, but can be costly and inaccessible for many clinicians. Alternatively, Wii balance-board technology is commercially accessible, and has previously shown high validity and reliability in mapping CoP deviation for older adults during static stance (Clark et al., 2010; Chang et al., 2013), but no evidence exists validating Wii balance-board technology and CoP deviation for people with IPD.

Our average PEDro score of 5.3 suggests that the studies included were of moderate methodological quality (Moseley et al., 2002). A lack of concealed allocation of participants, double blinding, and intention-to-treat analysis present as limitations of the methodology of the current exergaming literature. Furthermore, non-English studies were excluded here, and this presents as another limitation in the applicability of non-English exergaming literature. Lastly, only two of the studies included targeted people with IPD. Resultantly, our findings may not represent the larger community of people with IPD. Nevertheless, we deliberately included only RCT studies in an attempt to raise the standard of methodological quality. As such, while the IPD studies included were underwhelming, we have shown that there is a need for more robust, comprehensive studies on exergaming for people with IPD.

Our systematic review and meta-analysis demonstrates that exergaming can improve the balance of older adults. However, with the current available studies, the efficacy of exergaming cannot be sufficiently determined for people with IPD. While a non-favorable training effect was identified in two studies included in our meta-analysis, both studies implemented balance-specific training interventions for the control group. We interpret this to indicate exergaming has similar effects on balance as balance-specific training. Whether or not this training response is caused by physiological adaptation or neural adaptation is unknown, with the effects of exergaming on balance for people with IPD speculative to this point. As such, more robust RCT evidence is required to validate our findings.

## Conflict of Interest Statement

The authors declare that the research was conducted in the absence of any commercial or financial relationships that could be construed as a potential conflict of interest.

## Funding Source

This study was supported by the Central Research Grant Scheme (RM29471) and the School of Exercise and Nutrition Sciences, Deakin University. TR is supported by an Alfred Deakin Postdoctoral Fellowship. LJ is supported by the Australian Government’s Collaborative Research Networks program. MM is supported by a Labex NUMEV Fellowship (Digital and Hardware Solutions, Environmental and Organic Life Modeling, ANR-10-LABX-20).
